# Patient‐centred pathology reporting improves patient experience and understanding of disease in prostate cancer care

**DOI:** 10.1002/bco2.322

**Published:** 2024-02-06

**Authors:** Haidar Al Saffar, Alice Thomson, Jo‐Lynn S. Tan, Qiwei Wang, Emma Birch, Samantha Koschel, Elizabeth Medhurst, Dale Jobson, Sean Ong, Daniel A. Moon, Declan Murphy, Nathan Lawrentschuk

**Affiliations:** ^1^ Department of Genitourinary Cancer Surgery Peter MacCallum Cancer Centre Melbourne Victoria Australia; ^2^ St Vincent's Hospital, Melbourne Fitzroy Victoria Australia; ^3^ Melbourne Medical School, St Vincent's Hospital, Melbourne University of Melbourne Fitzroy Victoria Australia; ^4^ School of Public Health and Preventative Medicine Monash University Melbourne Victoria Australia; ^5^ EJ Whitten Prostate Cancer Research Centre Epworth Hospital Richmond Victoria Australia; ^6^ Department of Surgery (Urology) Epworth Hospital Richmond Richmond Victoria Australia; ^7^ Department of Surgery (Urology) Royal Melbourne Hospital Melbourne Victoria Australia

**Keywords:** Gleason grade group, pathology, patient centred, patient‐centred report, prostate cancer, radical prostatectomy, randomised control trial, surgery

## Abstract

**Introduction and Objectives:**

Patient‐centred (PC) and holistic care improves patient satisfaction and health outcomes. We sought to investigate the benefit of utilising a PC pathology report in patients undergoing radical prostatectomy (RP) for prostate cancer (PCa). Our study aimed to evaluate and compare patient understanding of their PCa diagnosis after RP, upon receiving either a standard histopathology report or a personalised and PC report (PCR). Moreover, we evaluated knowledge retention at 4 weeks after the initial consultation.

**Methods:**

We invited patients undergoing RP at three metropolitan Urology clinics to participate in our randomised controlled study. Patients were randomised to receive either a PCR or standard pathology report. Patient satisfaction questionnaires (Perceived Efficacy in Patient–Physician Interactions [PEPPI], Consultation and Relational Empathy [CARE] and Communication Assessment Tool [CAT]) and a knowledge test were conducted within 72 h of the initial appointment and again at 4 weeks. Accurate recollection of Gleason grade group (GGG) and extracapsular extension (ECE) were classified as ‘correct’. Baseline demographic data included age, education, marital and employment status, pre‐op prostate specific antigen (PSA) and clinical stage. Baseline data were tested for differences between groups using the Student's *t* test, chi‐squared test or Fisher's exact test depending on whether data were continuous, categorical or sparse. Comparison of correctly answered ‘knowledge’ questions was analysed using chi‐squared test. A significance level of *p* ≤ 0.05 was used.

**Results:**

Data from 62 patients were analysed (30 standard vs. 32 PCR). No significant differences in baseline demographics were found between groups. Both groups reported high levels of satisfaction with their healthcare experiences in all domains of patient–physician rapport, empathy and communication. There were no significant differences between groups in PEPPI (*p* = 0.68), CAT (*p* = 0.39) and CARE (*p* = 0.66) scores, at baseline and 4 weeks. Ninety‐three per cent of patients who received the PCR understood the report while 90% felt the report added to their understanding of their PCa. Regarding patient knowledge, the PCR group had significantly more correct answers on GGG and ECE as compared with the standard report group at baseline and 4 weeks (*p* < 0.001 and 0.001, respectively).

**Conclusions:**

Our findings demonstrate that PC pathology reports improve patient knowledge and understanding of their PCa that is retained for at least 4 weeks after initial receipt of results.

## 1 INTRODUCTION

Prostate cancer (PCa) is the most frequently diagnosed cancer in men worldwide and is the second leading cause of cancer‐related mortality in men.^1^ More than 1.4 million men are diagnosed with PCa every year, which for most is met with apprehension and anxiety when attempting to understand the implications of their disease and prognosis.^2^, ^3^

There is a trend in healthcare practice towards using universal health literacy precautions to provide understandable and accessible information to all patients, regardless of their literacy or education levels.^4^ Health literacy research supports breaking down information into small portions, avoiding medical jargon, using printed information and utilising visual aids to enhance patient understanding.^5^

Structured medical reporting has now been implemented across numerous sites to standardise reporting between radiologists.^6^ Examples of this includes radiological reporting using BIRADS (breast), PIRADS (prostate) and TIRADS (thyroid). Benefits of this type of reporting includes less time spent formulating the reports, reduced variability and improved clarity.^7^ Reports also show that referring specialists and radiologists had higher satisfaction ratings with structured reporting.^6^, ^8^ Standardised language is now being investigated and trialled as a measure to enhance communication and delivery of medical information to patients.^9^

It is widely acknowledged that patients are not traditionally the intended target audience for medical investigation reporting as health literacy rates remain mismatched with the language and terminology used, as well as lacking the specialist knowledge required to interpret the results.^10^ While patients have had increasingly uninhibited access to their records over the last 20 years, the excess of information presented in medical jargon may paradoxically make it more difficult for patients to truly understand their healthcare needs and thus navigate informed decision‐making.^11^ Failure to understand the information around their diagnosis can lead to anxiety about prognosis and outlook.^2^, ^12^

Patient‐centred (PC) pathology reporting has been investigated and found to be effective in improving patient understanding and experiences of receiving a diagnosis in bladder cancer and PCa.^13^, ^14^ In a study assessing the benefit after prostate biopsies, PC reports (PCRs) significantly improved Gleason score and number of positive cores recall rates.^13^ Moreover, 86% who received the PCR noted that the report helped them better understand their results and were in favour of this type of reporting always being available.^13^ The effect of PCR has also been studied in bladder cancer. Mossanen et al. utilised self‐efficacy scores, healthcare provider communication ratings, empathy scores and a knowledge test at 0 and 4 weeks.^14^ Patients who received the PCR demonstrated greater knowledge about their bladder cancer stage and preferred these reports were over the standard report.^14^

To our knowledge, the benefit of PCR in radical prostatectomy (RP) pathology has not been studied. Our study aimed to investigate the benefit of utilising a PCR in patients undergoing RP for PCa; evaluate and compare patient understanding of their PCa diagnosis post RP, upon receiving either a standard pathology report or a personalised PCR; and evaluate knowledge retention 4 weeks after receiving the initial post‐operative diagnosis.

We hypothesised that the use of PCRs, including a volumetric pictograph, will improve patient understanding and retention of knowledge about their PCa diagnosis. Moreover, the PCR will enable more effective communication between physicians and patients and can improve the patient experience.

## 2 OBJECTIVE, STUDY DESIGN AND METHODS

### 2.1 Study type and design and schedule

One hundred eight patients were prospectively recruited to participate in our randomised control trial (RCT) (Figure S1). Randomisation was carried out prior to patient recruitment from three high volume metropolitan urological practices. Patients were reviewed in the clinic within 4 weeks of surgery. Data were collected for baseline characteristics including age, highest level of education, first language, pre‐op prostate specific antigen (PSA), date of surgery, number of previous biopsies, marital status and employment status. Further pathological outcomes were also collected including positive margins, seminal vesicle invasion and lymphovascular invasion. Electronic surveys were then conducted within 72 h of the initial post‐operative follow‐up appointment and repeated at 4 weeks to assess patient perception of their healthcare experience and knowledge retention around their given diagnosis. As with the Mossanen et al. study on PCR in bladder cancer, patient focus groups identified the PC formats and language to convey elements and constructed a pilot PC pathology report and survey to be used in the study.

### 2.2 Inclusion criteria

Patients who underwent a RP for PCa and have yet to receive their post‐operative diagnosis and patients who attended in‐person or telehealth follow‐up appointments.

### 2.3 Exclusion criteria

Patients who only attended a telephone consult, patients who are non‐English speaking (NES) and patients with vision impairment limiting their ability to view any written/pictographic report.

### 2.4 Consent

Informed consent was obtained from all participants of the study, either in written form or, for patients who choose to complete and return the questionnaires, implied consent was assumed and accepted. Ethics approval was obtained (clinical study 67655). Participants were provided the opportunity to withdraw from the study at any point, while continuing to receive standard urological follow‐up and care.

### 2.5 Patient recruitment

Patients were recruited from three high volume metropolitan urological practices. All patients underwent robotic‐assisted RP at two metropolitan hospitals. A total of 62 patients (57%) were included in the final analysis. Of these, 32 patients were assigned to the PCR group and 30 to the standard reporting group.

### 2.6 Study outcomes

Outcomes were assessed using validated questionnaires including Perceived Efficacy in Patient–Physician Interactions (PEPPI),^15^ Consultation and Relational Empathy (CARE)^16^ and Communication Assessment Tool (CAT).^17^, ^18^, ^19^ Higher PEPPI, CARE and CAT scores indicate greater self‐efficacy, compassion and communication, respectively. A further knowledge test using a 5‐point Likert scale was carried out which assessed knowledge regarding surgical margins, ECE, nerve sparing status and tumour grade. These surveys were conducted electronically, and within 72 h of the initial post‐operative follow‐up appointment (baseline), and again at 4 weeks after initial review by the surgeon. Accurate recollection of Gleason grade group (GGG) and extracapsular extension (ECE) were classified as ‘correct’.

### 2.7 Generating the PCR

Operation and histopathology reports were reviewed by two Urology residents (Jo Tan and Haidar Al Saffar). Relevant data from the operation reports and formal pathology report were used to generate a PCR. For each PCR, data were entered into the Memorial Sloan Ketering Cancer Centre post‐radical prostatectomy nomogram (https://www.mskcc.org/nomograms/prostate/post_op) to generate disease prognosis including 15‐year PCa‐specific survival and recurrence‐free survival.

### 2.8 Statistical analysis

**FIGURE 1:**
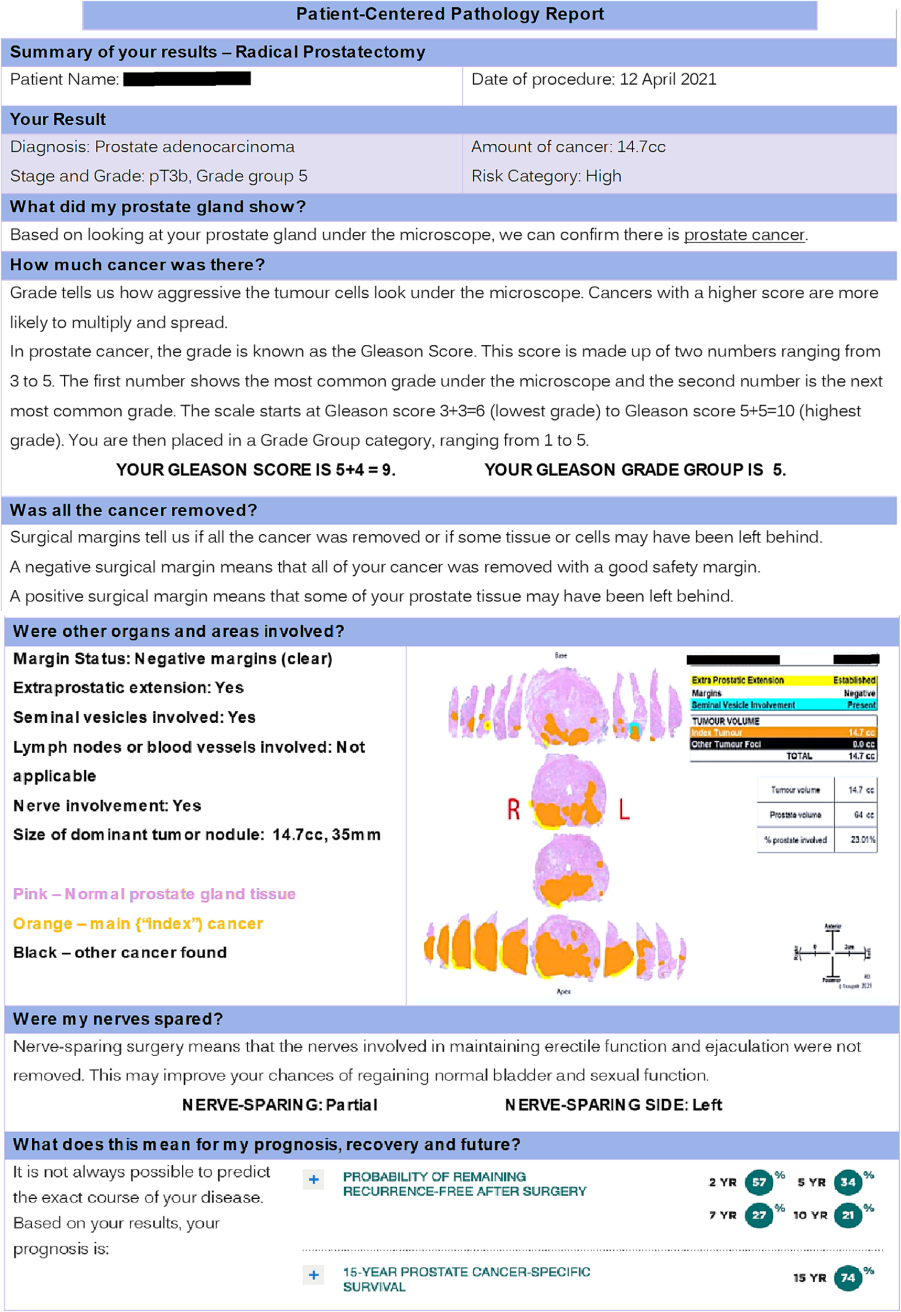
Sample patient‐centred report.

Demographic data were tested for differences between the standard report and PCR groups using the Student's *t* test, chi‐squared test or Fisher's exact test depending on whether the data were continuous, categorical or sparse. Differences in the number of correctly answered questions about pathology between groups were compared using chi‐squared test. *p* values of less than 0.05 were considered to indicate statistical significance. Statistical analyses were performed using GraphPad Prism version 8.0 (GraphPad Software, California, USA) (Figure 1).

## 3 RESULTS

### 3.1 Patient recruitment

Sixty‐two out of 108 patients (57%) were included in the final analysis (Figure S1). The remaining 46 (43%) who were not included in the final analysis included 21 (19%) patients who only completed one survey, 17 (16%) completed no surveys, four (4%) had incomplete surveys and four (4%) who withdrew from participating. RPs were conducted between March 2021 and February 2022.

### 3.2 Patient demographics

The mean age of the participants was 67 in the standard group and 65 in the PC group (*p* = 0.26). Forty‐two per cent held a bachelor degree with a further 22% with a graduate‐level degree, while 26% listed a high school degree or less as their highest level of education. Sixty‐one per cent of participants worked either full time or part time; the remaining 39% were retired. The average pre‐operative PSA level for standard medical report group was 9.17, with the patient‐centred report group having an average of 8.66. Most patients had GGG 2 disease. Pathological outcomes assessed included positive margin, seminal vesicle (SV) invasion and lymphovascular invasion rates. No significant baseline demographic differences were found between groups (Table 1).

**TABLE 1:** Demographics table for patients receiving the standard pathology report and PCR.

	Standard report (*n* = 30)	Patient‐centred report (*n* = 32)	*p* value
Mean age, years (SD)	67 (7.5)	65 (8.1)	0.26
Education, *n* (%)			0.47
Some college but no degree	5	3	
Less than high school degree	3	2	
High school degree	4	3	
Graduate degree	5	9	
Bachelor degree	11	15	
Associate degree	2	0	
First language at home, *n* (%)			0.99
English	29	31	
Other	1	1	
Gleason grade group			0.31
1	0	2	
2	14	15	
3	6	10	
4	3	2	
5	7	3	
Pathological outcomes			
Positive margin	4	3	0.62
Seminal vesicle invasion	5	3	0.39
Lymphovascular invasion	7	5	0.40

*Note*: There were no statistically significant differences in demographics between groups.

### 3.3 Patient satisfaction surveys

Both groups reported high levels of satisfaction with their healthcare experiences in all domains of patient–physician rapport, empathy and communication. There were no significant differences in PEPPI (0 weeks *p* = 0.61, 4 weeks *p* = 0.75), CAT (‘excellent’ rating scores at 0 weeks *p* = 0.30, 4 weeks *p* = 0.48) and CARE (0 weeks *p* = 0.78, 4 weeks *p* = 0.55) scores between groups (Table 2).

**TABLE 2:** PEPPI, CAT and CARE scores for both standard pathology report and PCR.

	Standard (*n* = 30)	Patient‐centred report (*n* = 32)	*p* value
PEPPI (efficacy), mean score (SD)			
Week 0	88 (15)	89 (11)	0.31
Week 4	87 (15)	89 (12)	0.15
CAT (communication), proportion of excellent ratings			
Week 0	47	53	0.12
Week 4	48	51	0.88
CAT (communication), mean score (SD)			
Week 0	4.6 (0.6)	4.7 (0.6)	0.39
Week 4	4.6 (0.6)	4.5 (0.7)	0.33
CARE (empathy), mean score (SD)			
Week 0	4.6 (0.6)	4.5 (0.7)	0.09
Week 4	4.5 (0.7)	4.4 (0.7)	0.60

*Note*: No statistically significant differences between groups were found using Student's *t* test.

### 3.4 Patient response towards the PCR

Ninety‐three per cent of patients with the PCR understood the report as compared with 80% in the standard group. Ninety per cent in the PCR group felt the report added to their understanding of their PCa compared with 83% in the standard group. Eighty‐four per cent in the PCR group noted that the report helped in their decision‐making moving forward compared with 73% in the standard group. Eighty‐one per cent in the PCR group felt confident about their PCa as compared with 66% in the standard group. Ninety‐three per cent of patients who received PCR understood the report while 90% felt the report added to their understanding of their PCa.

### 3.5 Patient recall

Accurate recollection of the GGG and ECE were classified as correct. At baseline, 12 patients (40%) provided correct answers while 18 (60%) had incorrect answers in the standard group. In comparison, the PCR group had 27 (84%) correct answers and five (16%) incorrect responses. This is statistically significant (*p* < 0.001).

**FIGURE 2:**
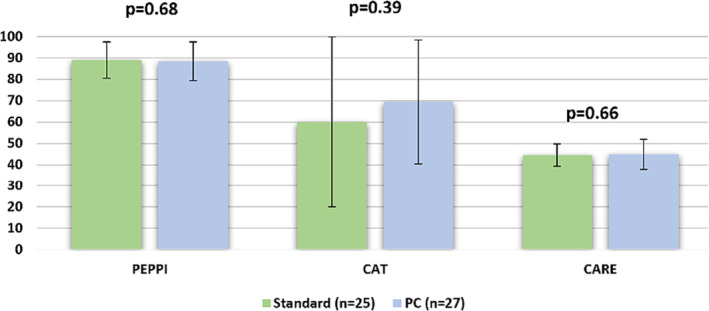
PEPPI, CAT and CARE scores. No statistically significant differences between groups were found using the Student's *t* test.

At Week 4, the standard group had 10 (33%) correct and 20 (67%) incorrect answers. This is in comparison with the PCR group which had 24 (75%) correct and eight (25%) incorrect responses. This was also statistically significant (*p* < 0.001) (Figure 2 and Table 3).

**TABLE 3:** Patient ability to accurately recall information about their Gleason grade and extracapsular extension for both standard pathology reports and patient‐centred reports.

Correctly answered questions about pathology, *n* (%)	Standard (*n* = 30)	Patient centred (*n* = 32)	*p* value
Week 0			<0.001
Correct	12 (40)	27 (84)	
Incorrect	18 (60)	5 (16)	
Week 4			0.001
Correct	10 (33)	24 (75)	
Incorrect	20 (67)	8 (25)	

*Note*: Patient‐centred reports had significantly more correctly answered questions compared with standard report at both Weeks 0 and 4 (*p* value <0.001).

## 4 DISCUSSION

Over the last 20 years, healthcare has made a considerable shift away from the paternalistic approach to a PC approach when relaying information, eliciting the patient's perspective and helping to guide decision‐making. We utilised a PCR to relay RP pathology and prognosis in the management of PCa and compared patient satisfaction and knowledge outcomes to the standard pathology group. Two studies published recently showed improved disease understanding and enhanced knowledge retention in bladder cancer and prostate biopsy reports.^13^, ^14^

In our study involving 62 patients, there was no widespread dissatisfaction with care (Figure 2). Patient self‐activation, empathy and communication were not significantly different between groups (Figure 2) which is consistent with previous reports.^13^, ^14^ Experienced urologists were involved in the project which is reflected by the effective communication skills whether patients received the PCR or not.

**FIGURE 3:**
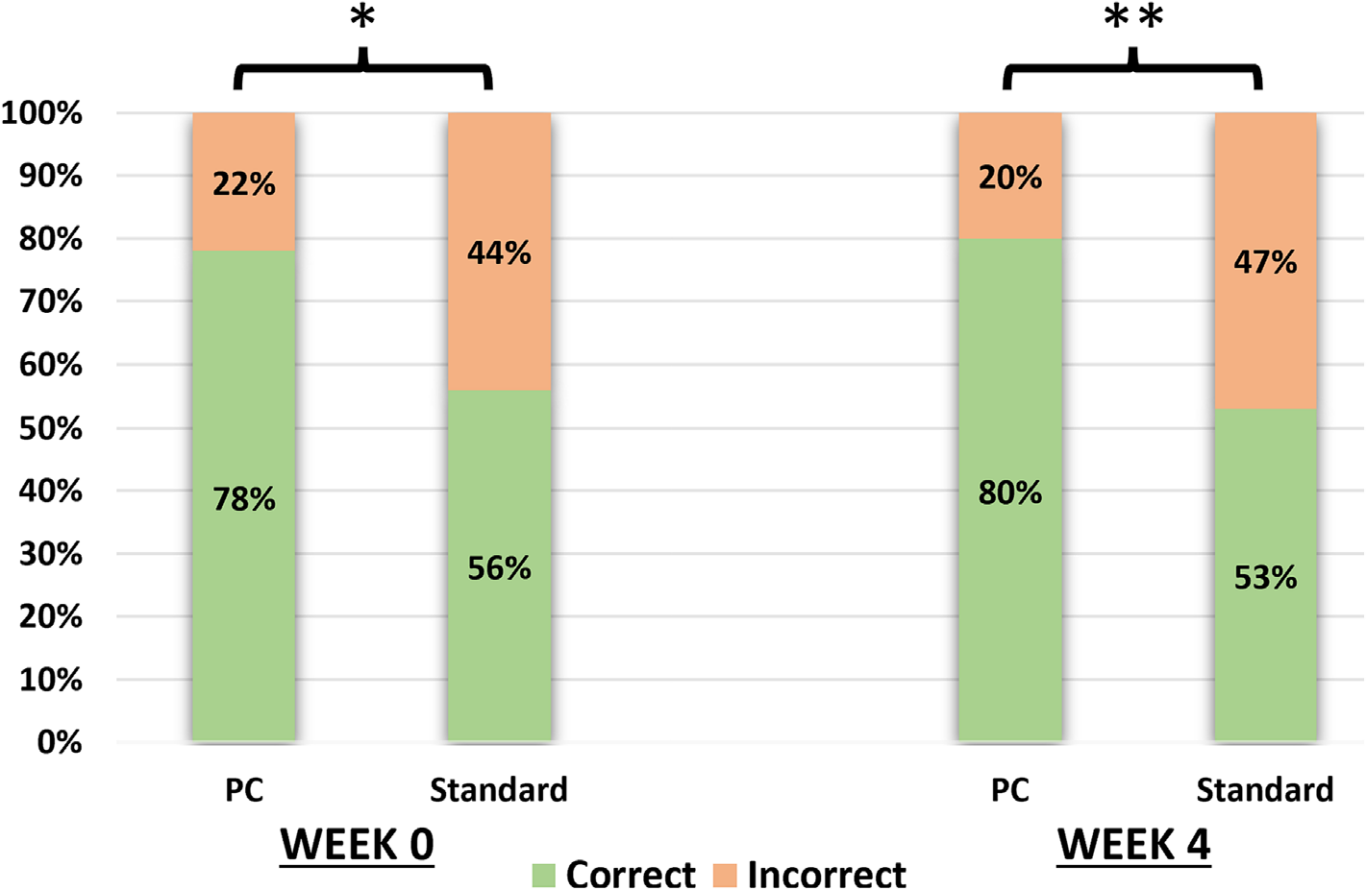
Patients ability to accurately recall information about their GGG and ECE for both standard pathology and PC reports. PC reports had significantly more correctly answered questions compared with standard report at both Weeks 0 and 4 (*p* value 0.01 and 0.004, respectively).

Regarding patient knowledge, the PCR group had significantly more correct answers on GGG and ECE, as compared with the standard report group, at baseline and 4 weeks (*p* < 0.001 and 0.001, respectively) (Figure 3).

Enhancing communication with patients about their diagnosis is associated with various positive health‐related results and emotional well‐being, treatment adherence and understanding of the treatment plan.^12^ RP procedures carry numerous risks, including the potential for long‐term urinary incontinence and erectile dysfunction. For patients having to cope with morbidity associated with the operation, keeping up with routine PSA screenings can pose a challenge for patients. PCR enhances this by improving the clinician–patient relationship and patient autonomy.

Approximately 30% of patients who undergo RP^20^, ^21^ and between 30% and 50% of men who receive radiotherapy may encounter biochemical recurrence within a decade after the therapy,^22^, ^23^ potentially leading to metastatic progression requiring further treatment. These may include salvage radiotherapy, hormonal therapy or chemotherapy. PCRs may be utilised to improve the patient–provider communication when disclosing new or recurrent cancer diagnoses. Early patient understanding of their pathology is vital to establish a knowledge base and make informed decisions about future therapies.

PC care is essential in providing care to patients with multiple conditions, which is particularly relevant to our ageing population undergoing treatment for oncological conditions. Improved patient understanding utilising PCR leads to improved trust in clinicians, adherence to follow‐up and reduced psychological distress associated with diagnosis.^24^ However, PCRs should be used to supplement clinical encounters and not be used as a substitute. We suggest a continuous process of PC interactions both before and after surgery.

Limitations of our study include a small sample size (62 patients) with a moderate dropout rate of 43%. Multiple reasons could have attributed to the dropout including inability to complete electronic surveys, time constraints and avoidance of health‐related anxiety associated with the surveys. Patients were requested to diligently fulfil two surveys with a time gap of 4 weeks, a task that might have proven excessively challenging as a result of their ongoing surgical recovery, potential lapses in memory or other unforeseen circumstances. Moreover, as patients were expected to interpret the pathology report, only English‐speaking patients were included in the study. Additionally, all patients received care in the private‐hospital system. This observation potentially accounts for the fact that they predominantly originated from affluent backgrounds and possessed tertiary education, as depicted in Table 1.

Clinicians were not blinded to patient randomisation which could have influenced the interaction. Furthermore, consultation duration was not measured. External counselling, such as information provided by the family doctor or sought by the patient online, was also not measured or controlled for in this study. Patients presenting with initial or recurrent diagnoses may have possessed differing levels of pre‐existing knowledge prior to their clinical encounter.

The research focused exclusively on the period immediately following prostatectomy. Additionally, due to ethical constraints within the study's scope, there was no further exploration of delayed follow‐up or data collection regarding the incidence of recurrence and subsequent adjuvant therapy.

While our study shows a significant benefit in this selected patient group who are mostly from affluent backgrounds (Figure 3 and Table 1), one may propose that the benefit towards the general population could be even greater which could be explored further in PCa and other medical conditions. Future research may focus on these specific groups when receiving PCR that is targeted at education level and provided in the patient's first language. Additionally, future research should control for factors such as clinician experience and external patient counselling, as patients may have greater benefit from receiving PCR when being counselled by less‐experienced doctors.

Despite limitations, this study serves as a model for future PCR studies. Results must be validated in a multicentre setting with a diverse PCa population to ensure its generalisability, including patients attending public hospital and patients of varying education levels.

Once validated, the broad implementation of the PCR into real‐world practice may serve to strengthen PC decision‐making. To allow for efficient, accurate generation of patient‐centred pathology reports, an electronic program to generate reports can be established and utilised by the reporting pathologist.

Healthcare provision has shifted to a PC approach which has been shown to improve health outcomes. Patients undergoing investigations and management of cancer care are particularly vulnerable; thus, effective communication is vital to improving patient understanding and satisfaction when receiving their diagnosis and prognosis. Our RCT demonstrates that after undergoing a RP, patients preferred to receive the PCR which in turn improved knowledge and understanding of their PCa that is retained for at least 4 weeks. Further large‐scale randomised studies investigating the benefit of PCR in urological cancer and other medical conditions are needed.

## AUTHOR CONTRIBUTIONS

**Haidar Al Saffar:** Data collection; data analysis; writing—original draft. **Alice Thomson:** Data collection; data analysis; writing—original draft. **Jo‐Lynn S. Tan:** Data collection; data analysis; writing—original draft. **Qiwei Wang:** Writing and data collection. **Emma Birch:** Data collection. **Samantha Koschel:** Data collection. **Elizabeth Medhurst:** Data collection. **Dale Jobson:** Statistics. **Sean Ong:** Data analysis; writing—original draft. **Daniel A. Moon:** Conceptualization; methodology; data collection. **Declan Murphy:** Conceptualization; methodology; data collection. **Nathan Lawrentschuk:** Conceptualization; methodology; data collection; writing—review and editing.

## CONFLICT OF INTEREST STATEMENT

The authors declare no conflicts of interest.

## Supporting information


**Figure S1:** CONSORT flow diagram for the study.
**Figure S2:** Patient response towards their pathology report between the standard and the PCR group. There were no statistically significant differences between groups.
**Figure S3:** Patient knowledge questionnaire.
