# Supplementary material for: Patient‐centred pathology reporting improves patient experience and understanding of disease in prostate cancer care
Source: BJUI Compass. 2024 Feb 6;5(4):497–505. doi: 10.1002/bco2.322 (PMC11019249; doi:10.1002/bco2.322)
Supplement: Supplementary file 1 — Figure S1: CONSORT flow diagram for the study. Figure S2: Patient response towards their pathology report between the standard and the PCR group. There were no statistically significant differences between groups. Figure S3: Patient knowledge questionnaire. [file BCO2-5-497-s001.docx]

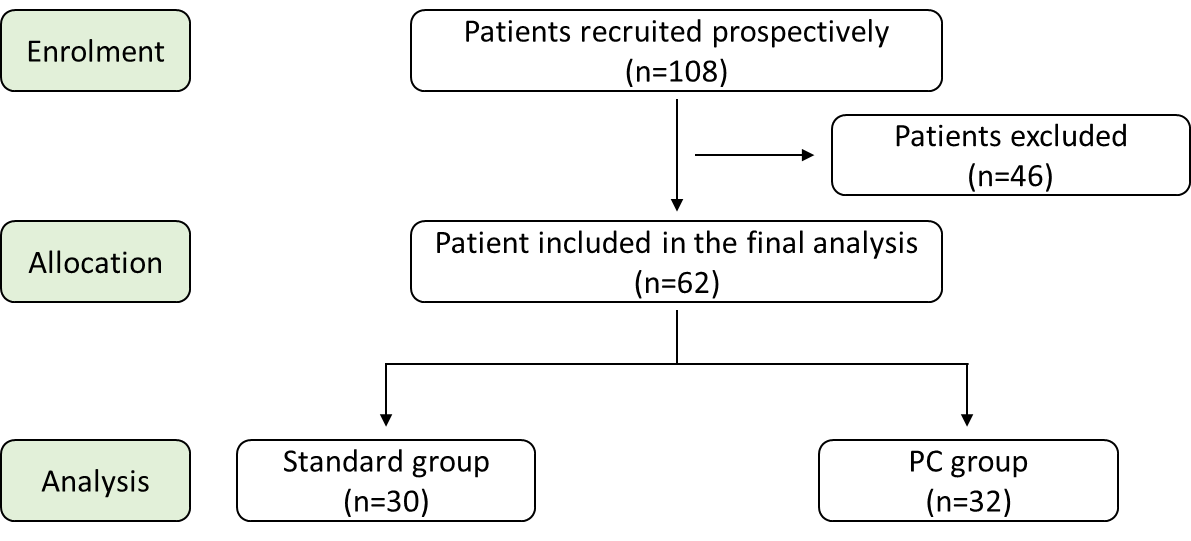


**Supplementary Figure 1**: CONSORT flow diagram for the study.


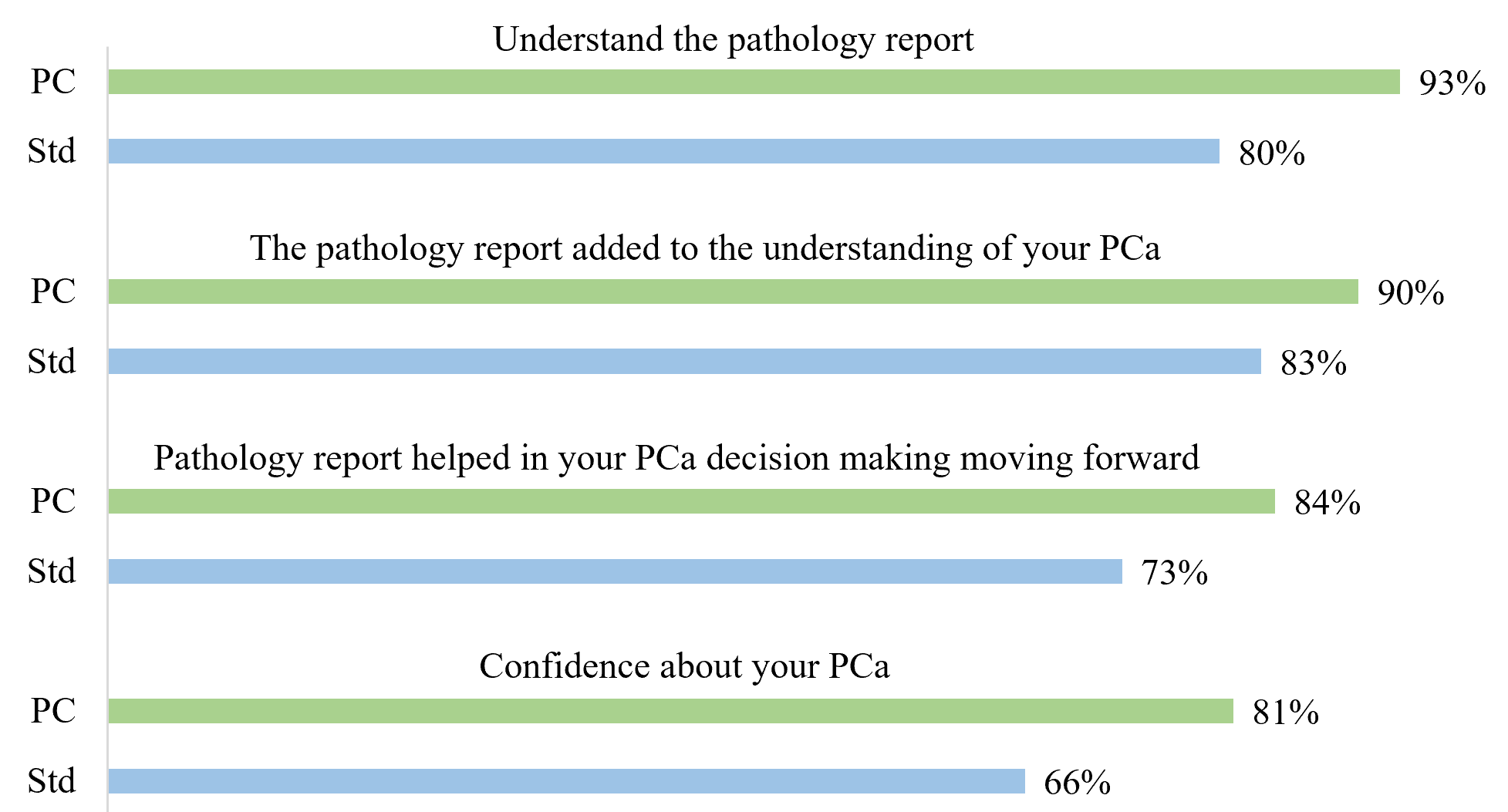


**Supplementary Figure 2**: Patient response towards their pathology report between the standard and the PCR group. There were no statistically significant differences between groups.


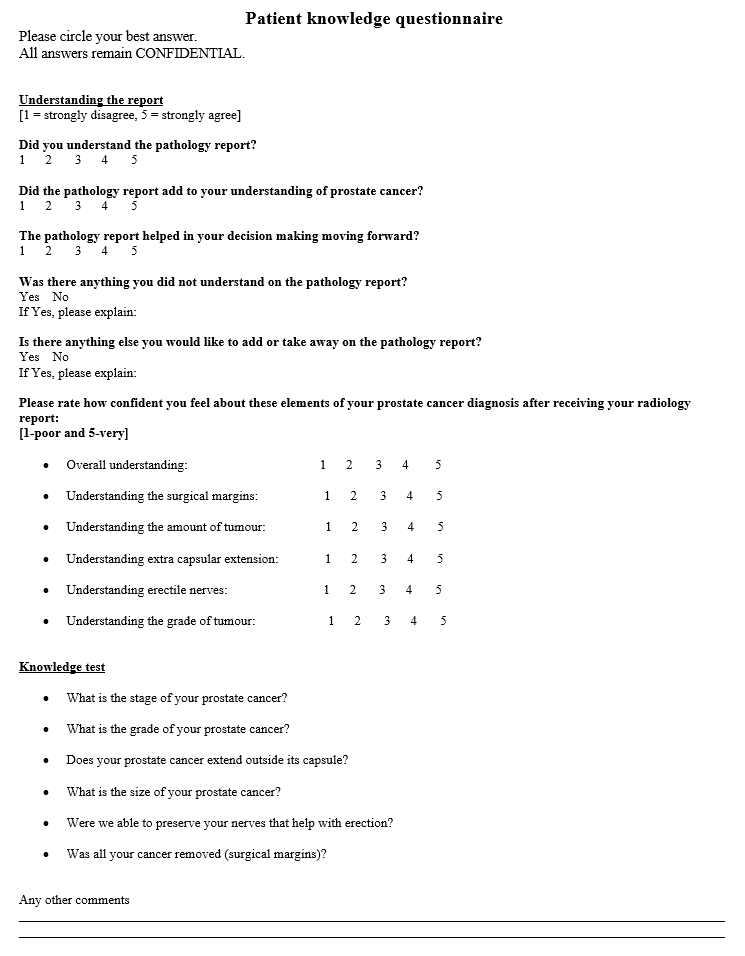
**Supplementary Figure 3**: Patient knowledge questionnaire.
